# Risk Factors for Recurrence after Robot-Assisted Radical Hysterectomy for Early-Stage Cervical Cancer: A Multicenter Retrospective Study

**DOI:** 10.3390/cancers12113387

**Published:** 2020-11-16

**Authors:** Jordi Ponce, Sergi Fernandez-Gonzalez, Antonio Gil-Moreno, Pluvio J. Coronado, Jesús De la Rosa, Henrique Nabais, Ginés Hernández, Anna Taltavull, Juan Gilabert-Estelles, Sergio Martínez-Román, Manel Barahona, Marc Barahona, María Ángeles Martínez-Maestre

**Affiliations:** 1 Department of Gynecology, University Hospital of Bellvitge (IDIBELL), L’Hospitalet de Llobregat, 08907 Barcelona, Spain; jponce@bellvitgehospital.cat (J.P.); mbarahona@bellvitgehospital.cat (M.B.); 2 Department of Gynecologic Oncology, Hospital Universitari Vall d’Hebron, 08035 Barcelona, Spain; agil@vhebron.net; 3 Instituto de Salud de la Mujer, Hospital Clínico San Carlos, Madrid, IdISSC, Universidad Complutense, 28040 Madrid, Spain; pcoronado@gmail.com; 4 Department of Gynecology, Hospital Universitario de Basurto, 48013 Bilbao, Spain; jhrosafer@hotmail.com; 5 Department of Gynecology, Champalimaud Foundation, 1400-038 Lisbon, Portugal; henrique.nabais@fundacaochampalimaud.pt; 6 Department of Gynecology, Hospital Universitario Quirónsalud, 28223 Madrid, Spain; gines.hernandez@quironsalud.es; 7 Department of Gynecology, Hospital Universitari Josep Trueta, 17007 Girona, Spain; ataltavull.girona.ics@gencat.cat; 8 Department of Gynecology, Hospital General Universitario de Valencia, University of Valencia, 46014 Valencia, Spain; gilabert_juaest@gva.es; 9 Department of Gynecology, Hospital Universitari Germans Trias i Pujol, Badalona, 08916 Barcelona, Spain; smartinezro.germanstrias@gencat.cat; 10 Department of Gynecology, University Hospital of Puerto Real, 11510 Cádiz, Spain; manuel.barahona@uca.es; 11 Department of Gynecology, Hospital Universitario Virgen del Rocio, 41013 Sevilla, Spain; martinez.maestre@hotmail.com

**Keywords:** early-stage cervical cancer, robotic surgery, radical hysterectomy, oncological outcome, recurrence

## Abstract

**Simple Summary:**

In 239 women with early-stage cervical cancer (≤IB1 or IIA1, FIGO 2009) undergoing robot-assisted radical hysterectomy in Spanish and Portuguese centers between 2009 and 2018, the overall survival rate was 94.1% after a median follow-up of 51 months. Recurrence was diagnosed in 26 patients. In the multivariate analysis, independent risk factors for recurrence were tumor size > 20 mm, adenocarcinoma as histological type, presence of positive pelvic lymph nodes, tumor grades 2 and 3, and not performing sentinel lymph node biopsy. The present oncological and surgical results surpassed the target of quality indicators in cervical cancer proposed by the European Society of Gynecology Oncology. When selecting a robot-assisted surgical approach to perform radical hysterectomy in the surgical treatment of primary early-stage cervical cancer, it is recommended to take into account the tumor grade and histological type, results of the sentinel lymph node biopsy, and the size of the tumor.

**Abstract:**

This retrospective analysis aimed to assess the risk factors for recurrence in patients diagnosed with early-stage cervical cancer (≤IB1 or IIA1, FIGO 2009) undergoing robot-assisted radical hysterectomy in Spain and Portugal between 2009 and 2018. A second primary objective was to audit the oncological outcomes according to quality indicators (QI) proposed by the European Society of Gynecology Oncology (ESGO). The study population included 239 women. After a median follow-up of 51 months, recurrence occurred in 26 patients (10.9%). Independent factors for recurrence were clinical tumor size > 20 mm (hazard ratio (HR) 2.37), adenocarcinoma as histological type (HR 2.51), positive pelvic lymph nodes (HR 4.83), tumor grade 2 (HR 4.99), tumor grade 3 (HR 8.06), and having not performed sentinel lymph node biopsy (SLNB) (HR 4.08). All 5 QI selected were surpassed by our results. In patients with early-stage cervical cancer undergoing robotic radical hysterectomy, clinicians should be aware that tumor grade 2 and 3, tumor size > 20 mm, adenocarcinoma, positive pelvic nodes, and lack of performance of SLNB are risk factors for recurrence. Fulfillment of QI targets of the ESGO might be considered as an objective oncological outcome indicator supporting the minimally invasive approach for early-stage cervical cancer treatment.

## 1. Introduction

Since the Food and Drug Administration (FDA) approval of the da Vinci^®^ robot for gynecologic surgery in 2005, robot-assisted minimally invasive surgery (MIS) has become increasingly common in gynecological oncology [1,2,3,4]. Observational studies published in the literature before 2018 concluded that either traditional laparoscopic [5,6,7] or robot-assisted laparoscopic [8,9,10,11,12] approaches provided similar oncological outcomes to open surgery. In 2018, the Laparoscopic Approach to Cervical Cancer (LACC) trial [13], a phase 3 trial comparing MIS (laparoscopic or robotic) radical hysterectomy with open radical hysterectomy in women with early-stage cervical cancer revealed a disease-free survival (DFS) rate at 4.5 years that was lower with MIS than with open surgery (86.0% vs. 96.5%) and a lower 3-year rate of overall survival (OS) (93.8% vs. 99.0%). However, of the patients who were assigned to MIS, 84.4% underwent laparoscopy and 15.6% robot-assisted surgery. Based on these unexpected results and data of two further observational studies [14,15], in 2019 the European Society of Gynecological Oncology (ESGO) established the open approach as the gold standard for radical hysterectomy [16] and recommended to reserve MIS for highly-specialized centers in gynecological surgery. In January 2020, ESGO also published quality indicators (QI) for the surgical treatment of cervical cancer [17] with the intention to audit and improve clinical practice in an easy and practicable way. Nevertheless, whether results of laparoscopic and robotic surgery can be superimposed remains a matter of debate.

Cancer of the cervix ranks fourth in the list of the most common malignancies and causes of death in women globally [18]. In 2018, 570,000 diagnoses of cervical cancer worldwide and about 311,000 deaths in women were estimated from the disease [19]. However, the incidence of cervical cancer is decreasing in Southern European countries [20], and in Spain, an incidence of 2000 cases diagnosed per year was estimated [21]. About half of women with cervical cancer are diagnosed at early stages (≤IB1 or IIA1) and standard treatment includes radical hysterectomy with pelvic lymphadenectomy and/or sentinel lymph node biopsy.

The primary objective of this study was to assess the risk factors for recurrence in all women with early-stage cervical cancer who underwent robotic surgery in the departments of gynecology of all centers of the Iberian Peninsula (9 hospitals in Spain and 1 hospital in Portugal) in which da Vinci^®^ technology was available. The second primary objective was to audit the oncological and surgical results according to recommendations for cervical cancer surgery proposed by the ESGO [17]. The secondary objective was to validate the prognostic value of the 2018 International Federation of Gynecology and Obstetrics (FIGO) staging system [22] in our series.

## 2. Results

A total of 263 women with a clinical diagnosis of early-stage (IA1, IA2, IB1, IIA1) cervical cancer underwent robot-assisted radical hysterectomy during the study period. Twenty-four patients were upstaged after surgical resection (stage IB2, *n* = 9; ≥ IIB, *n* = 15) (FIGO 2009) [23] and were excluded from the analysis of recurrence, but the audit of QI was performed in the whole series of 263 patients.

### 2.1. Clinical and Pathological Characteristics

The mean age of the study population (*n* = 239) was 48 years. As shown in Table 1, salient features included median tumor size ≤ 20 mm 69.1% of the patients, squamous cell carcinoma in 58.9%, stage IB1 in 80.3%, negative nodal status in 89.5%, tumor grade 2 in 52.9%, histological tumor size ≤ 20 mm in 62.8%, and stromal infiltration less than one-third of tissue core in 61.2%. There were 21 patients with stage IA1 cervical cancer, but only two of them were aged < 35 years; no fertility-sparing procedures were performed in these cases.

### 2.2. Surgical Procedures and Adjuvant Treatment 

As shown in Table 2, the most common surgical procedure was type C1 radical hysterectomy, which was performed in 67.9% of the patients. Other salient data were use of uterine manipulator in 68.6% of patients, negative surgical margins in 97.5%, and sentinel lymph node biopsy (SLNB) was not performed in 50.2%.

Major intraoperative complications were recorded in 11 (4.2%) patients, postoperative complications in 25 (9.5%), and long-term complications in 6 (2.4%) (Table S1).

### 2.3. Recurrence and Risk Factors

After a median follow-up of 51 months, recurrence was diagnosed in 26 patients (≤IB1 or IIA1 stage), with a recurrence rate of 10.9%. Vaginal recurrences were diagnosed in 8 patients, pelvic in 8, lymph nodes in 3, and systemic in 3. After 2 years of follow-up, recurrences were present in 14 patients (recurrence rate 5.9%). In the univariate analysis, age, clinical and MRI tumor size, histological type, tumor grade, histological tumor size, and stromal infiltration were risk factors for recurrence (Table 1). In 19 (73.1%) of the 26 patients with recurrence, SLNB was not performed with a hazard ratio (HR) of 1.47 (95% confidence interval (CI) 0.59–3.67) (*p* = 0.406) (Table 2). Five recurrences had negative SLNB, and one had micrometastasis. In the multivariate analysis, independent risk factors for recurrence were clinical tumor size > 20 mm, adenocarcinoma and other histological types, positive lymph nodes, tumor grades 2 and 3, and not having performed SLNB (Table 3).

### 2.4. Oncological Outcomes

The OS rate was 94.1% (225/239) and the DSF was 89.1% (213/239). The cancer specific mortality rate was 4.6% (11/239). There were statistically significant differences in DFS at 5 years when patients were grouped according to risk factors for recurrence (Table 4).

**Figure 1:**
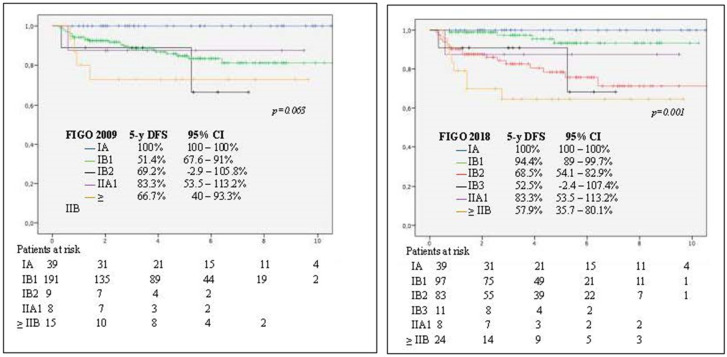
Kaplan–Meier analysis of disease-free survival (DFS) at 5 years. Differences between use of International Federation of Gynecology and Obstetrics (FIGO) 2009 (log-rank test, *p* = 0.063) (left panel) and FIGO 2019 (log-rank test, *p* = 0.001) (right panel) staging systems.

The prognostic FIGO 2018 classification was found to be more accurate than FIGO 2009 classification system (Figure 1).

Finally, the result obtained in 231 patients (stages ≤ IB1) in terms of recurrence and oncological outcome were similar to those observed in all 263 women who underwent robot-assisted radical hysterectomy during the study period (Table S2).

### 2.5. Audit of Quality Indicators

Five of six quality outcome indicators of the ESGO for surgical treatment of cervical cancer [17] were fulfilled, except for a higher percentage of patients receiving adjuvant therapy for a stage pT1b1 pN0 disease (Table 5).

## 3. Discussion

In recent years, numerous studies compared oncological outcomes (OS and DFS) between open approach and MIS in women with cancer of the cervix. However, as far as we are aware, this is the first study which was specifically designed to audit the oncological and surgical outcomes among the national (Spain and Portugal) population of early-stage cervical cancer who underwent robotic surgery. Risk factors for recurrence after robot-assisted radical hysterectomy (ordered from the highest to the lowest risk) were tumor grade 3, tumor grade 2, positive lymph nodes, histological aggressive subtypes, adenocarcinoma, not having performed SLNB, and clinical tumor size > 20 mm. Moreover, our findings confirm that the FIGO 2018 staging system [22] is a better prognosticator of outcome as compared with the FIGO 2009 system [23] because of the inclusion of a new cut-off for tumor size (2 cm) and lymph node involvement.

Recent studies evaluated the oncological outcomes in patients with cervical tumors ≤ 20 mm [24,25,26]; in particular, the study of Anchora et al. [27] compared patients with 2009 FIGO stage from IA1 with lymphovascular space invasion to IB1/IIA1 treated by open or laparoscopic surgery and found that laparoscopic and open approaches showed superimposable DFS. A tumor diameter of 20 mm was considered as the choice of surgical approach, with recommendation of open surgery for tumors > 20 mm and either laparoscopic or open surgery for tumors < 20 mm [27]. However, in a retrospective study of 815 women with tumor size ≤ 20 mm, in whom radical hysterectomy was performed by MIS in 255 cases and open surgery in 560 cases, the MIS approach was noted to be independently associated with a higher likelihood of recurrence (adjusted HR, 6.31; 95% CI 1.24–31.9) [28]. Candidates in our study we eligible women for robotic surgery from the beginning of the initiation of robotic surgery programs up to 2018, but given the retrospective analysis of data, outcomes in tumors sizes ≤ 20 mm and > 20 mm were evaluated. Interestingly, clinical tumor size > 20 mm was a predictor of recurrence but showed the lowest HR as compared to other variables. Therefore, pathological factors such as histological subtype and tumor grade should also be considered when deciding to perform minimally invasive radical hysterectomy for early-stage cervical cancer [29]. In a nationwide population-based cohort study carried out in Sweden with 864 women with cervical cancer stage IA1-IB undergoing radical hysterectomy in 2011–2017 (open surgery 236, robotic 628), tumor size and grade 3 were significant risk factors for DFS but not for OS, although risk factors for recurrence were not analyzed [30].

In addition, in case of selection of MIS, ESGO [16] recommends to collect data prospectively and to take extreme precautions to avoid tumor spillage [31], including ligation of the upper vagina before the laparoscopic procedure and to abandon uterine manipulator, considered as a risk factor for recurrence. The SUCCOR study published by Chiva et al. [32] observed that patients who underwent MIS without the use of a uterine manipulator had similar disease-free-survival to the open surgery group (HR, 1.58; 95% CI, 0.79 to 3.15; *p* = 0.20). By contrast, in our study, there was a non-significant 4% increase in recurrence among patients in whom the uterine manipulator was used.

A further interesting aspect of the study was the prognostic impact of not having performed SLNB, with a statistically significant likelihood of recurrence of 4.08 (95% CI 1.54–10.78). Sentinel node biopsy is a valuable procedure for a more accurate diagnosis of nodal involvement and for reducing lymphatic morbidity as an alternative option to standard pelvic lymphadenectomy. In this respect, an international validation study (SENTICOL III) of SLNB in women with early-stage cancer of the cervix with last accrual scheduled in 2021 and last follow-up in 2026, will provide conclusive data of 3-year DFS after SLN biopsy alone or SLN+ pelvic lymphadenectomy [33]. The intraoperative diagnosis of macrometastases is crucial to avoid overtreatment for patients with advanced stages who would benefit from chemoradiotherapy [34].

As mentioned above, the present results of robotic surgery were evaluated according to the QI for cervical cancer surgery proposed by the ESGO [17] in January 2020. Of note, our results of indicators related to the quality of surgical procedures were all beyond the proposed targets. On the other hand, those results are inferior to the open surgery reported of LACC [13] where the 4.5 years DFS was 96.5%. Should the QI be revised on the basis of this cut-off in order to increase the level of excellence in gynecological oncology? In case of fulfilling the QI, is either approach permitted? Results of the currently ongoing international randomized multicenter RAAC trial (robot-assisted approach to cervical cancer) [35] will provide important data of recurrence-free survival at 5 years between women treated with robot-assisted laparoscopic surgery versus laparotomy for early-stage cervical cancer. In the meantime, the MIS approach should be performed by highly experienced surgical centers.

Limitations of the study include the retrospective design and small sample size due to difficulties related to the absence of strict centralization of cervical cancer cases associated with the low prevalence of cancer of the cervix as compared to studies carried out in other countries [35]. However, selection bias was reduced by selecting all consecutive eligible women undergoing robotic surgery for early-stage cervical cancer based on the total number of departments of gynecology in Spanish and Portuguese hospitals with availability of robotic technology during the study period. Although this study may be considered regionally specific, the novel aspect is the assessment of results obtained with robot-assisted radical surgery in early-stage cervical cancer patients as the only surgical approach.

## 4. Materials and Methods

### 4.1. Study Design and Participants

All patients diagnosed with early-stage cervical cancer undergoing robot-assisted radical hysterectomy in Spain and Portugal were eligible for inclusion in a retrospective cohort multicenter study. Inclusion criteria were as follows: infiltrating cervical cancer diagnosed on biopsy, to be candidates for radical surgery according to FIGO 2009 classification [23], and to be operated on by robotic surgery from the beginning of the initiation of robotic surgery programs up to 2018. All cases were collected from 10 tertiary care hospitals, 9 in Spain and 1 in Portugal. The primary objective of the study was to assess risk factors for recurrence. A second primary objective was to audit the surgical results according to recommendations of quality indicators for cervical cancer surgery proposed by the ESGO [17]. Secondary objectives were to assess oncological outcomes and to validate the FIGO 2018 classification. All patients gave written informed consent for the surgical procedure.

### 4.2. Surgical Procedure and Adjuvant Treatment

The type of radical hysterectomy was left at the discretion of the attending surgeon and categorized according to the Querleu–Morrow classification [36] and the use of uterine manipulator. All operations were performed using the da Vinci^®^ surgical system. Pelvic lymph node status was evaluated by means of SLNB and/or pelvic lymphadenectomy depending on the surgeon’s criterion. Positive pelvic lymph nodes gathered all patients with positive lymph nodes including micrometastases or isolated tumor cells after SLNB ultrastaging. The SLNB was detected by technetium ± blue dye or indocyanine green (ICG) or ICG alone. Macrometastasis or micrometastasis and isolated tumor cells were analyzed in the SLNB ultrastaging procedure. When the presence of macrometastases was diagnosed in frozen section intraoperatively, radical hysterectomy was not performed. Postoperative adjuvant treatment was indicated depending on the center’s protocol for the management of cervical cancer, taking into account the FIGO stage, risk factors, or positive margins. Adjuvant treatment included external-beam radiotherapy (50 Gy/5 weeks) ± brachytherapy ± chemotherapy (cisplatin 40 mg/m^2^ every week during external radiation therapy).

### 4.3. Oncological Outcomes and Audit of Quality Indicators

Recurrence of the disease was diagnosed by the combination of clinical, radiological, and histological findings. The time elapsed from the date of radical hysterectomy to diagnosis of recurrence was used to define DFS, and the date of operation to death from any cause to define OS. The audit of oncological outcomes was performed by comparison of the results obtained with recommended targets of QI proposed by the ESGO [17]. From 15 QI specifically designed for the analysis of surgical or treatment outcomes, 5 surgical QI and 1 adjuvant treatment QI were selected.

### 4.4. Data Collection

Clinical and pathological variables were collected and included age, body mass index (BMI, kg/m^2^), clinical tumor size by inspection or ultrasound, size measured by MRI, tumor histology, FIGO stage after surgery, histologic tumoral size, surgical margins, presence of invasion of the lymphovascular space (LVSI), infiltration of the stroma, and tumor grade. LVSI was diagnosed when malignant cells were present in epithelial-lined spaces of the cervical stroma. The depth of stromal infiltration from the basement membrane was measured in millimeters and expressed as thirds of the total stromal width. The pathologist measured the size of the cervical tumor as the largest diameter on the cone and gross tissue samples. Complications included grade II–IV complications recorded intraoperatively [37] during the first 30 postoperative days (early complications) and after 30 days of surgery (late complications) [38].

### 4.5. Statistical Analysis

Categorical variables are reported as absolute numbers and percentages. Mean values and standard deviation (SD) or median values and range (minimum–maximum) were used to express quantitative variables. Categorical variables were analyzed with the chi-square test of the Fisher’s exact test, whereas continuous variables were analyzed with the Student’s *t* test. Risk factors for recurrence of cervical cancer were determined by Cox proportional hazards regression analysis, with hazard ratios (HRs) with 95% confidence intervals (CIs) as the measures of risk. Oncological outcomes, OS and DFS, were calculated with the Kaplan–Meier method, with differences in the probability of survival analyzed with the log-rank test. The date of the last follow-up was the censored date for patients without events. The IBM SPSS Version 23.0 and R 3.0.3 program was used for statistical analysis. A *p* value of < 0.05 was considered statistically significant.

## 5. Conclusions

In this study of women with early-stage cervical cancer undergoing robot-assisted radical hysterectomy, risk factors for recurrence were tumor grade, adenocarcinoma and aggressive histological subtypes, clinical tumor size, positive lymph nodes, and lack of performance of intraoperative SLNB. Therefore, when considering robotic surgery for the management of ≤ IB1 cervical cancer, careful selection of patients would include tumors < 20 mm, squamous cell carcinoma histological type, tumors grade 1, and to perform SLNB intraoperatively in order to reduce the likelihood of recurrence. The quality of surgical outcomes obtained in the present series was beyond recommended quality targets for cervical cancer surgery.

## Figures and Tables

**Table 1:** Clinical and Pathological Data.

Variables	All Patients (*n* = 239)	Recurrence (*n* = 26)	Hazard Ratio (95% CI)	*p* Value
Age, years, median (range)	48 (25–81)	51 (34–81)	1.05 (1.01–1.09)	0.007
BMI, median (range)	26 (17–59)	27 (19–35)	1.01 (0.96–1.07)	0.716
Clinical tumor size, mm, median (range)	15 (0–40)	25 (0–40)	1.07 (1.03–1.1)	<0.001
≤20 mm, *n* (%)	159 (69.1)	11 (44)	1	
>20 mm, *n* (%)	71 (30.9)	14 (56)	3.2 (1.45–7.09)	0.004
MRI tumor size, mm, median (range)	15 (0–40)	26 (3–40)	1.05 (1.01–1.09)	0.013
≤20 mm, *n* (%)	136 (66.7)	8 (42.1)	1	
>20 mm, *n* (%)	68 (33.3)	11 (57.9)	2.87 (1.15–7.14)	0.023
Stage of disease, *n* (%)				
IA1	21 (8.8)	0	NA	
IA2	18 (7.5)	0	NA	
IB1	192 (80.3)	25 (96.2)	NA	
IIA1	8 (3.3)	1 (3.8)	NA	
Histological type, *n* (%)				
Squamous cell carcinoma	141 (58.9)	10 (38.5)	1	
Adenocarcinoma	89 (37.2)	13 (50)	1.97 (0.84–4.48)	0.108
Other	9 (3.8)	3 (11.5)	6.29 (1.72–23.02)	0.005
Nodal status, *n* (%)				
Negative	214 (89.5)	22 (84.6)	1	
Positive	11 (4.6)	4 (15.4)	4.32 (1.48–12–59)	0.023
Lymphovascular space involvement, *n* (%)	42 (17.6)	7 (26.9)	1.75 (0.74–4.19)	0.2
Tumor grade, *n* (%)				<0.001
1	74 (31.1)	2 (7.7)	1	
2	126 (52.9)	14 (53.8)	4.73 (1.08–20.85)	0.04
3	38 (16)	10 (38.5)	10.49 (2.29–47.73)	0.002
Histological tumor size, mm, median (range)	18 (0–40)	25 (3–40)		0.001
≤20 mm, *n* (%)	150 (62.8)	10 (38.5)	1	
>20 mm, *n* (%)	89 (37.2)	16 (61.5)	2.89 (1.31–6.37)	0.006
Stromal infiltration, *n* (%)				<0.001
<1/3	112 (61.2)	0	NA	
1/2 to 2/3	43 (23.5)	7 (53.8)	1	
<2/3	28 (15.3)	6 (46.2)	1.33 (0.45–3.97)	0.6

CI: confidence interval; BMI: body mass index; NA; not applicable; chi-square test or Fisher’s exact test for the comparison of categorical variables, and Student’s *t* test for continuous variables.

**Table 2:** Surgical and Adjuvant Characteristics.

Variables	All Patients (*n* = 239)	Recurrence (*n* = 26)	Hazard Ratio (95% CI)	*p* Value
Type of radical hysterectomy, *n* (%)				0.016
Trachelectomy	1 (0.49)	0	NA	
A	14 (5.9)	0	NA	
B1	52 (21.9)	1 (3.8)	0.12 (0.01–0.90)	0.037
B2	9 (3.8)	1 (3.8)	0.66 (0.09–4.86)	0.68
C1	161 (67.9)	24 (92.3)	1	
Use of uterine manipulator, *n* (%)				
No	75 (31.4)	6 (23.1)	1	
Yes	164 (68.6)	20 (76.9)	1.47 (0.59–3.67)	
Surgical margins status, *n* (%)				
Negative	233 (97.5)	24 (92.3)	1	
Positive	6 (2.5)	2 (7.7)	3.3 (0.79–14.28)	0.099
Sentinel lymph node, *n* (%)				0.013
Biopsy performed	119 (49.8)	7 (26.9)	1	
Biopsy not performed	120 (50.2)	19 (73.1)	1.47 (0.59–3.67)	0.040
Adjuvant treatment, *n* (%)				0.110
None	178 (74.5)	16 (61.5)	1	
Yes	61 (25.5)	10 (38.5)	1.76 (0.79–3.88)	0.161
Chemoradiotherapy, *n* (%)	20 (8.4)	5 (19.2)		
Radiotherapy ± brachytherapy, *n* (%)	41 (17.2)	5 (19.2)		

CI: confidence interval; NA; not applicable; chi-square test or Fisher’s exact test for the comparison of categorical variables, and Student’s *t* test for continuous variables.

**Table 3:** Results of Multivariate Analysis.

Predictive Factors	Hazard Ratio (95% CI)	*p* Value *
Clinical tumor size > 20 mm	2.37 (1.05–6.07)	0.038
Histological type		
Adenocarcinoma	2.51 (1.03–6.07)	0.042
Other (mixed, sarcoma, clear cell, glassy cell)	4.36 (1.14–16.61)	0.031
Tumor grade		
2	4.99 (1.11–22.45)	0.036
3	8.06 (1.68–38.6)	0.009
SLNB not performed	4.08 (1.54–10.78)	0.005
Positive nodal status	4.83 (1.40–16.63)	0.012

CI: confidence interval; SLNB: sentinel lymph node biopsy; * Wald chi-square test.

**Table 4:** Disease-Free Survival and Risk Factors for Recurrence.

Variables	Disease–Free Survival, % (95% CI)	*p* Value *
Clinical tumor size		
≤20 mm	89.3 (82.6–95.9)	0.002
>20 mm	69.9 (54.3–85.4)	
Histological type		
Squamous cell carcinoma	90.1 (84.3–95.9)	0.008
Adenocarcinoma	76.4 (63.5–89.4)	
Other	60 (24.9–95.1)	
Nodal status		
Negative	83.5 (76.3–90.7)	0.003
Positive	52.9 (19.4–86.5)	
Tumor grade		
1	96.4 (91.6–101.3)	0.001
2	79.3 (67.6–91)	
3	69.2 (53.4–85.1)	
Sentinel lymph node biopsy		
Performed	87.1 (75.7–98.6)	0.049
Not performed	79.6 (71–88.3)	

* log-rank test.

**Table 5:** European Society of Gynecology Oncology (ESGO) Quality Indicators Related to Surgical Procedure for Cervical Cancer.

Outcome Indicator	Target Value	Present Series
QI 9—Urological fistula rate within 30 postoperative days after radical parametrectomy	≤3%	0%
QI 10—Proportion of patients after primary surgical treatment who have clear vaginal (invasive disease) and parametrial margins	≥97%	97.3% (213/219)
QI 11—Proportion of patients with a stage T1b disease T-upstaged after surgery	<10%	9.1% (24/263)
QI 12—Recurrence rate at 2 years in patients with a stage pT1b1 with negative lymph nodes after surgery	<10%	5.1% (9/175)
QI 13—Proportion of patients with a T1 disease treated by primary surgery who underwent lymph node staging	≥98%	99.6% (1/218)
QI 15—Proportion of patients receiving adjuvant chemoradiotherapy after a primary surgical treatment for a stage pT1b1 pN0 disease	<15%	28.1% (44/182)

## Supplementary Materials

The following are available online at https://www.mdpi.com/2072-6694/12/11/3387/s1: Table S1: Major surgical complications, Table S2: Oncological outcomes & Recurrences.
