# Supplementary material for: Risk Factors for Recurrence after Robot-Assisted Radical Hysterectomy for Early-Stage Cervical Cancer: A Multicenter Retrospective Study
Source: Cancers (Basel). 2020 Nov 16;12(11):3387. doi: 10.3390/cancers12113387 (PMC7696424; doi:10.3390/cancers12113387)
Supplement: Supplementary file 1 [file cancers-12-03387-s001.zip › cancers-981857-supplementary.docx]

Risk Factors for Recurrence after Robot-Assisted Radical Hysterectomy for Early-Stage Cervical Cancer: A Multicenter Retrospective Study

Jordi Ponce, Sergi Fernandez-Gonzalez, Antonio Gil-Moreno, Pluvio J. Coronado,
Jesús De la Rosa, Henrique Nabais, Ginés Hernández, Anna Taltavull,
Juan Gilabert-Estelles, Sergio Martínez-Román, Manel Barahona, Marc Barahona ^1^ and María Ángeles Martínez-Maestre

**Table S1.** Major Surgical Complications in 263 Patients.

| **Complications** | **Number of patients (%)** |
| --- | --- |
| Intraoperative, grade II–IV * | 11 (4.2) |
| Urinary | 10 (3.8) |
| Intestinal | 1 (0.4) |
| Postoperative, grade II–IV ^†^ | 25 (9.5) |
| Urinary | 7 (2.7) |
| Pelvic hematoma | 5 (1.9) |
| Intestinal | 4 (1.5) |
| Pelvic abscess | 4 (1.5) |
| Vaginal dehiscence | 2 (0.8) |
| Fever | 3 (1.1) |
| Long-term complications, grade II–IV ^†^ | 6 (2.4) |
| Lymphedema | 1 (0.4) |
| Chronic pelvic pain | 3 (1.1) |
| Hernia | 1 (0.4) |
| Urgent urinary incontinence | 1 (0.4) |

* CLASSIC classification; † Clavien-Dindo classification.

**Table 2.** Recurrence and oncological outcome in 231 patients with stage ≤ IB1 as compared to all 263 women who underwent robot-assisted radical hysterectomy during the study period.

| **Variables** | **Stage ≤ IB1 (*n* = 231), follow-up** | | **All patients (*n* = 263), follow-up** | |
| --- | --- | --- | --- | --- |
|  | **2 years** | **51 months (median)** | **2 years** | **51 months (median)** |
| Recurrence, *n* (%) | 14 (6.1) | 25 (10.8) | 20 (7.6) | 32 (12.2) |
| Vaginal | 5 (2.6) | 7 (2.7) | 6 (2.3) | 8 (3) |
| Pelvic | 3 (1.6) | 6 (2.3) | 3 (1.1) | 6 (2.3) |
| Lymph nodes | 3 (1.6) | 5 (2) | 5 (1.9) | 7 (2.7) |
| Systemic | 3 (2.1) | 7 (3) | 6 (2.3) | 11 (4.2) |
| Overall survival, *n* (%) | 227 (98.3) | 217 (93.9) | 257 (97.7) | 246 (93.5) |
| Cancer specific survival, *n* (%) | 3 (1.3) | 11 (4.8) | 5 (1.9) | 13 (4.9) |

| 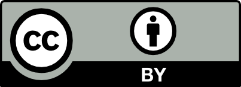 | © 2020 by the authors. Licensee MDPI, Basel, Switzerland. This article is an open access article distributed under the terms and conditions of the Creative Commons Attribution (CC BY) license (http://creativecommons.org/licenses/by/4.0/). |
| --- | --- |
